# Boosting the Dehydrogenation Properties of LiAlH_4_ by Addition of TiSiO_4_

**DOI:** 10.3390/ma16062178

**Published:** 2023-03-08

**Authors:** Nurul Yasmeen Yusnizam, Nurul Amirah Ali, Noratiqah Sazelee, Mohammad Ismail

**Affiliations:** Energy Storage Research Group, Faculty of Ocean Engineering Technology and Informatics, Universiti Malaysia Terengganu, Kuala Nerus 21030, Malaysia; nurulyasmeen.yusnizam@gmail.com (N.Y.Y.); nurulllamirah@gmail.com (N.A.A.); atiqahsazelee19@gmail.com (N.S.)

**Keywords:** LiAlH_4_, TiSiO_4_, hydrogen storage, dehydrogenation properties

## Abstract

Given its significant gravimetric hydrogen capacity advantage, lithium alanate (LiAlH_4_) is regarded as a suitable material for solid-state hydrogen storage. Nevertheless, its outrageous decomposition temperature and slow sorption kinetics hinder its application as a solid-state hydrogen storage material. This research’s objective is to investigate how the addition of titanium silicate (TiSiO_4_) altered the dehydrogenation behavior of LiAlH_4_. The LiAlH_4_–10 wt% TiSiO_4_ composite dehydrogenation temperatures were lowered to 92 °C (first-step reaction) and 128 °C (second-step reaction). According to dehydrogenation kinetic analysis, the TiSiO_4_-added LiAlH_4_ composite was able to liberate more hydrogen (about 6.0 wt%) than the undoped LiAlH_4_ composite (less than 1.0 wt%) at 90 °C for 2 h. After the addition of TiSiO_4_, the activation energies for hydrogen to liberate from LiAlH_4_ were lowered. Based on the Kissinger equation, the activation energies for hydrogen liberation for the two-step dehydrogenation of post-milled LiAlH_4_ were 103 and 115 kJ/mol, respectively. After milling LiAlH_4_ with 10 wt% TiSiO_4_, the activation energies were reduced to 68 and 77 kJ/mol, respectively. Additionally, the scanning electron microscopy images demonstrated that the LiAlH_4_ particles shrank and barely aggregated when 10 wt% of TiSiO_4_ was added. According to the X-ray diffraction results, TiSiO_4_ had a significant effect by lowering the decomposition temperature and increasing the rate of dehydrogenation of LiAlH_4_ via the new active species of AlTi and Si-containing that formed during the heating process.

## 1. Introduction

Concerns about the energy crisis and the environment have led to an increase in the proportion of renewable energy sources in the energy system, such as wind, hydro, and other types. The supply and utilization times are typically out of sync and have some geographical restrictions. The proper secondary energy must be selected to match them, and hydrogen is a promising alternative to meet the demands for clean and sustainable energy technologies [1]. Hydrogen energy is a perfect substitute for petroleum because of its high energy density and lack of carbon emissions [2,3,4,5,6,7].

Hydrogen can be stored in a variety of ways for application purposes. As of now, compressed gas has been the most popular technique. It can also be kept as a liquid at extremely low temperatures. Other methods of storing hydrogen are via the solid-state method through physisorption and chemisorption [8]. Storing hydrogen via the solid-state method is regarded as the most promising method. Based on the US Department of Energy (DOE), by 2025, the fuel cell materials should store 5.5 wt% (gravimetric) and 40 g L^−1^ (volumetric) of hydrogen [9]. Complex hydride has emerged as the most promising medium for solid-state hydrogen storage based on the DOE target due to its high hydrogen storage capacity. Additionally, based on previous research, storing hydrogen is promising in metal or complex hydrides via chemisorption. This technique involves absorbing and storing hydrogen in a metal powder made of either pure metal or an alloy. The metal/complex hydride material generates heat when hydrogen is absorbed into it, and heat is required for the metal/complex hydride to generate hydrogen again. As a result of the hydrogen’s strong bonding with the metal in some materials, it requires extreme temperatures, more than 400 °C for magnesium, as an example [10,11]. This is a disadvantage of the metal/complex hydride storage method [12,13,14]. Among metal or complex hydride materials, one of the best potential candidates is said to be lithium alanate (LiAlH_4_) [15,16,17,18,19,20]. LiAlH_4_ is thought to be a promising material for storing hydrogen in solid-state form as it has a greater capacity to store hydrogen compared to other complex hydrides. Table 1 compares the properties of LiAlH_4_ with other complex hydrides.

LiAlH_4_ decomposes in three steps, as shown in Equations (1)–(3),

LiAlH_4_ → 1/3Li_3_AlH_6_ + 2/3Al + H_2_ (about 5.3 wt% of H_2_ liberated) (1)

Li_3_AlH_6_ → 3LiH + Al + 3/2H_2_ (about 2.6 wt% of H_2_ liberated) (2)

LiH + Al → LiAl + 1/2H_2_ (about 2.6 wt% of H_2_ liberated) (3)

However, its application is hampered by its high dehydrogenation temperature, slow dehydrogenation kinetics, and reversibility issues. According to previous studies [19,26,27,28], these challenges were addressed through several modifications, such as particle size reduction utilizing the ball milling method and doping with a catalyst. Some examples of doping with catalysts in the literature [19,29,30,31,32,33,34,35] include doping with metal halides, metal oxides, and carbon-based additives.

Complex metal oxide additives, especially secondary metal oxides, are a newly emerging research area to further enhance LiAlH_4_’s dehydrogenation abilities [36,37,38]. The study of the catalysis of Al_2_TiO_5_ on the dehydrogenation properties of LiAlH_4_ demonstrated that the onset temperatures dropped significantly after milling with 5 wt% Al_2_TiO_5_ [39]. The hydrogen began to be liberated at about 90 °C and 137 °C for both steps, respectively. Meanwhile, Zhai et al. [36] investigated how MnFe_2_O_4_ affected LiAlH_4_’s dehydrogenation properties and discovered that the temperature at which decomposition began was about 70 °C lower than it would have been otherwise. Moreover, the research conducted by Ismail et al. [40] proved that the onset desorption temperature of the two stages of the LiAlH_4_ was reduced to 80 °C and 120 °C, respectively, when doped with SrTiO_3_, as opposed to the as-obtained LiAlH_4_. The LiAlH_4_ dehydrogenation kinetics were also enhanced as the SrTiO_3_-doped LiAlH_4_ composite could liberate about 3.0 wt% of hydrogen at 90 °C in 20 min as opposed to the undoped composite’s 0.2 wt% in the same time frame.

However, more research is necessary to determine whether other secondary metal oxide-based catalysts could lower the decomposition temperature and improve LiAlH_4_ dehydrogenation kinetic efficiency. In this study, we have used another secondary metal oxide to boost the dehydrogenation properties of LiAlH_4_. To the best of our knowledge, no research has been conducted on enhancing LiAlH_4_’s ability to store hydrogen using TiSiO_4_ as a catalyst.

## 2. Materials and Methods

Sigma Aldrich (Burlington, MA, USA) provided LiAlH_4_ (purity 95.0%) and TiSiO_4_ (purity 99.8%) powders that were used without any changes. Every stage of the sample preparation process, including loading and weighing, was completed in an MBraun Unilab glove box to avoid oxidation. The sample (LiAlH_4_ + 10 wt% TiSiO_4_) was then directly milled in an NQM-0.4 planetary ball mill for 1 h (15 min milling, followed by three cycles of 2 min rest between rotations) at a rate of 400 rpm.

A Sievert-type apparatus (Advanced Materials Corporation, Pittsburgh, PA, USA) was used to study the onset dehydrogenation temperature and the kinetic property for the liberation of H_2_ from the undoped LiAlH_4_ and the TiSiO_4_-added LiAlH_4_ composite. The sample was heated from room temperature to 250 °C at a heating rate of 5 °C/min in a vacuum environment for the onset dehydrogenation temperature characterization. To determine the kinetics of H_2_ liberation, the sample was heated at a constant temperature of 90 °C while being exposed to an H_2_ pressure of 1.0 atm. Differential scanning calorimetry (DSC) from Setline STA: Simultaneous Thermal Analysis (SETARAM) was used to investigate the thermal properties of the samples. The DSC measurements were run at heating rates of 15 °C/min, 20 °C/min, 25 °C/min, and 30 °C/min. The argon gas flow rate was set to 50 mL/min, and the temperature range for this measurement was set to 300 °C. The microstructure of the sample before and after the milling process was examined using scanning electron microscopy (SEM; JEOL JSM 6360LA). The phase structure of the sample before and after the ball mill and after the dehydrogenation process was studied using an X-ray diffractometer (XRD, Rigaku Miniflex, Tokyo, Japan) and Fourier transform infrared (IR, Shimadzu Tracer-100, Tokyo, Japan).

## 3. Results and Discussion

**Figure 1:**
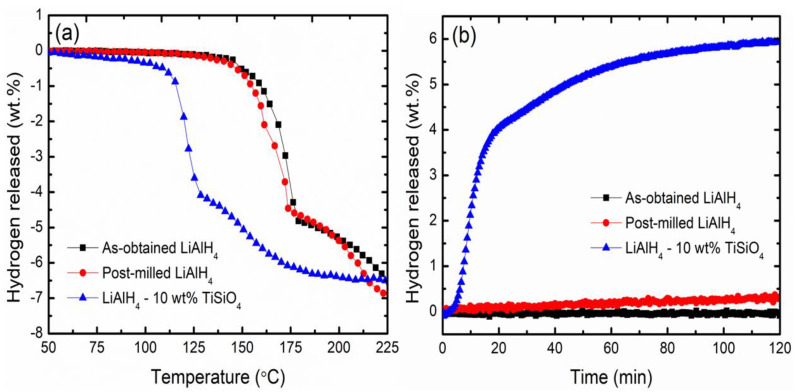
(**a**) TPD curve of as-obtained LiAlH_4_, post-milled LiAlH_4,_ and LiAlH_4_ added with 10 wt% TiSiO_4_ at 250 °C and (**b**) the dehydrogenation kinetics performance at a constant temperature of 90 °C for the added and undoped composites.

Figure 1 depicts the results of the research on the onset thermal dehydrogenation and dehydrogenation kinetics processes for both added and undoped composites (LiAlH_4_ and 10 wt% TiSiO_4_ systems). Two distinguishing features of each composite’s dehydrogenation reaction are shown on the graph. As shown in Figure 1a, at about 146 °C, the as-obtained LiAlH_4_ has initiated the liberation of hydrogen, and further heating has led to the beginning of a second stage dehydrogenation process at about 180 °C. After 1 h of the LiAlH_4_ milling process, the first and second steps’ onset dehydrogenation temperatures drop to about 144 °C and 174 °C, respectively. This result demonstrates how the ball milling technique insignificantly influenced the dehydrogenation temperature of LiAlH_4_. Moreover, doping 10 wt% of TiSiO_4_ drastically lowered the decomposition temperatures for the two steps at 92 °C and 128 °C. Adding TiSiO_4_ as a catalyst proved to enhance the dehydrogenation performance of LiAlH_4_.

Figure 1b shows the isothermal dehydrogenation kinetics at 90 °C for doped and undoped samples. Less than 1.0 wt% of hydrogen was liberated in 80 min by the undoped sample. Under the same conditions, the hydrogen’s liberate capacity increased significantly to 5.7 wt% after 10 wt% TiSiO_4_ was added. As a result, the TiSiO_4_-added LiAlH_4_ sample desorbs at a rate that is, on average, 5 to 6 times higher than the undoped LiAlH_4_. According to this result, the catalyst addition of TiSiO_4_ improved the kinetics of LiAlH_4_ dehydrogenation. The outcome shows that the kinetics of LiAlH_4_ dehydrogenation were improved by the catalyst doping, as previous studies stated [15,41,42].

**Figure 2:**
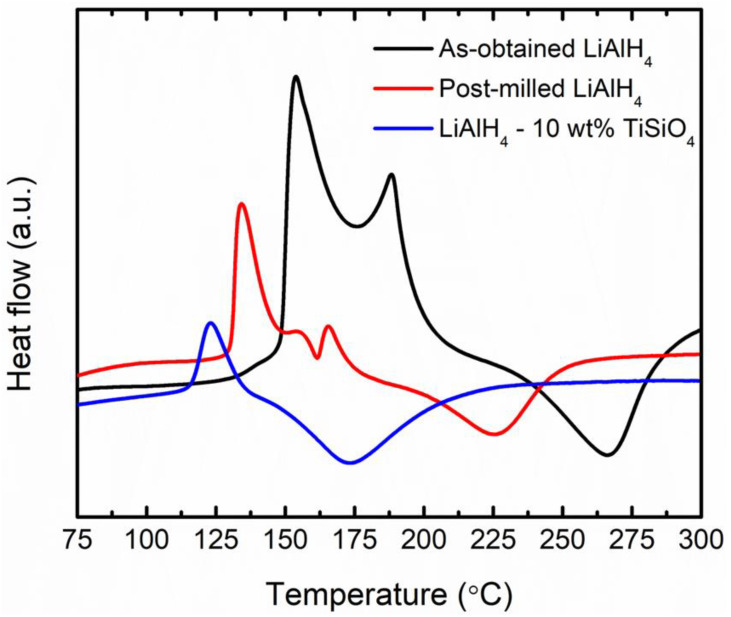
DSC data recorded at a rate of 15 °C/min for as-obtained LiAlH_4_, post-milled LiAlH_4_, and LiAlH_4_ added with 10 wt% TiSiO_4_.

The catalytic effects of TiSiO_4_ on the thermal properties of LiAlH_4_ were validated using DSC measurements on added and undoped LiAlH_4_. Temperatures ranging from 25 °C to 300 °C, with an argon flow rate of 50 mL/min, were used for the investigation. Figure 2 displays the DSC curves for added and undoped LiAlH_4_ at a rate of 15 °C/min. The thermal characteristics of as-obtained LiAlH_4_ have four peaks. Two are exothermic, while the other two are endothermic. The interaction of hydroxyl impurities with LiAlH_4_’s surface caused the first exothermic peak to appear at 150 °C, while the melting of LiAlH_4_ caused the first endothermic peak to appear at 175 °C [37,40,43,44]. In the meantime, the dehydrogenation of liquid LiAlH_4_ causes the second exothermic peak (190 °C). Li_3_AlH_6_ decomposition (265 °C) was responsible for the second endothermic peak. These second exothermic and endothermic peaks were assigned to Equations (1) and (2), respectively. These similar peaks also occurred to the post-milled LiAlH_4_, but at lower temperatures.

The thermal event of LiAlH_4_ is lowered from four to two after the addition of TiSiO_4_. The exothermic event peak at around 120 °C seems to correspond to the decomposition of LiAlH_4_ (Equation (1)), and the endothermic event peak at around 176 °C is most likely to correspond to the decomposition of Li_3_AlH_6_ (Equation (2)). The first endothermic event associated with LiAlH_4_ melting has vanished from the DSC curve of TiSiO_4_-doped LiAlH_4_. The disappearance of the melting event is most likely owing to the fact that the decomposition temperature of the first stage of the doped sample is lower than the melting temperature of post-milled LiAlH_4_.

**Figure 3:**
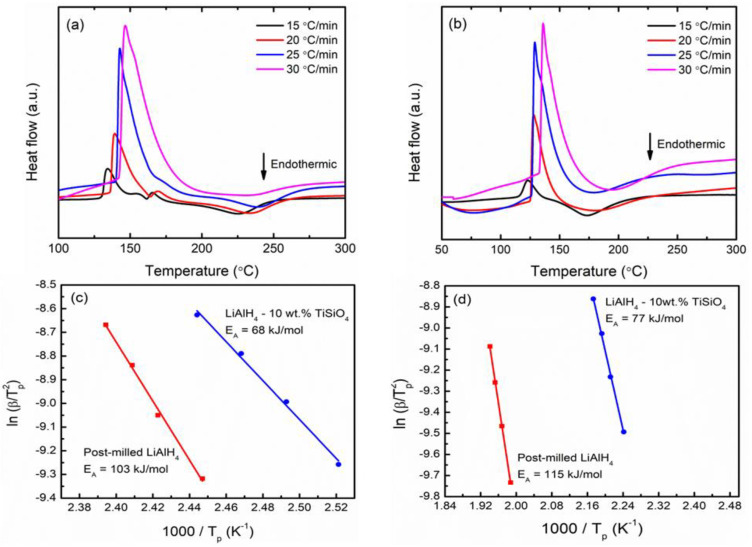
DSC dehydrogenation curves at different heating rates for (**a**) post-milled LiAlH_4_, (**b**) LiAlH_4_–10 wt% TiSiO_4_ and Kissinger’s plot for the (**c**) first and (**d**) second dehydrogenation stage of post-milled LiAlH_4_ and LiAlH_4_ added with 10 wt% TiSiO_4_.

Different heating rates were measured using DSC to examine the impact of TiSiO_4_ addition on the activation energy (*E*_A_) for hydrogen desorbed from LiAlH_4_. The DSC curves for the added and undoped LiAlH_4_ samples at various heating rates (15, 20, 25, and 30 °C/min) are shown in Figure 3a,b. The Kissinger equation used to calculate the *E*_A_ values of the dehydrogenation process of the 1 h milled LiAlH_4_ and LiAlH_4_–10 wt% TiSiO_4_ composite is as follows:

ln [*β/T_p_*^2^] = −*E_A_*/*RT_p_* + *A* (4)

where *R* is the gas constant, *A* is a linear constant, *T_p_* is the peak temperature on the DSC dehydrogenation curves, *E_A_* is the activation energy, and *β* is the DSC heating rate. Consequently, the *E_A_* was determined using the graph’s slope, ln $\left[\beta /T_{p}^{2}\right]$ vs. 1000/*T_p_*. The Kissinger plots for the dehydrogenation of LiAlH_4_ (1st stage) and the dehydrogenation of Li_3_AlH_6_ (2nd stage) for both composites are shown in Figure 3c,d. Based on the slopes, *E*_A_ for the post-milled LiAlH_4_ are 103 kJ/mol (1st stage dehydrogenation) and 115 kJ/mol (2nd stage dehydrogenation), respectively. Meanwhile, the *E_A_* for the first and second stages of dehydrogenation, respectively, are reduced to 68 kJ/mol and 77 kJ/mol for the added system with 10 wt% TiSiO_4_.

**Figure 4:**
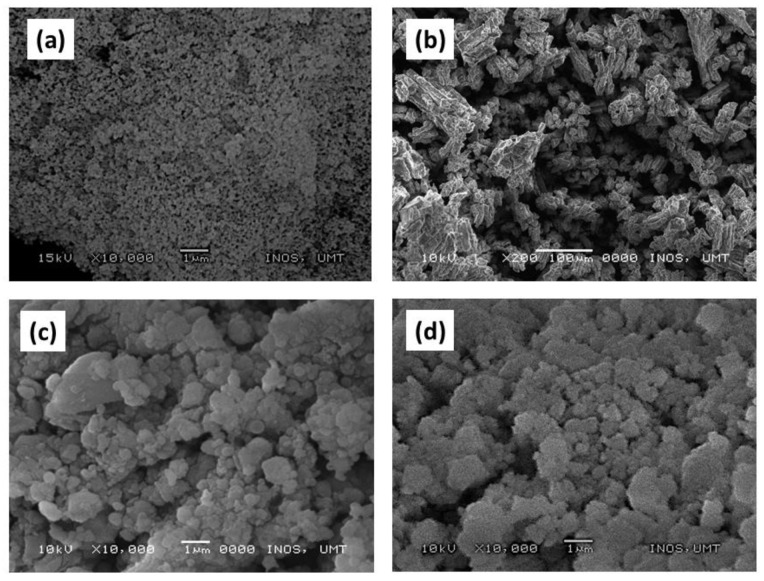
The morphological structures of (**a**) as-obtained TiSiO_4_, (**b**) as-obtained LiAlH_4_, (**c**) post-milled LiAlH_4,_ and (**d**) LiAlH_4_ added with 10 wt% TiSiO_4_.

Figure 4 shows the morphology differences between the TiSiO_4_, added, and undoped LiAlH_4_ samples. Figure 4a shows the morphology for the as-obtained TiSiO_4_, which is a fine particle. Figure 4b depicts the as-obtained LiAlH_4_’s morphology, which is described as rough and asymmetrical in shape. The particle size of LiAlH_4_ was smaller but aggregated and inhomogeneous after a 1 h milling process (Figure 4c). The particle size of the 10 wt% TiSiO_4_-doped LiAlH_4_ sample (Figure 4d) has shrunk and is less aggregated compared to post-milled LiAlH_4_. The 10 wt% TiSiO_4_-added LiAlH_4_ composite has a larger surface area due to the smaller particle sizes. Previous research has shown that surface modification significantly improves the hydrogen storage properties of metals and complex hydride materials [34,45,46,47].

**Figure 5:**
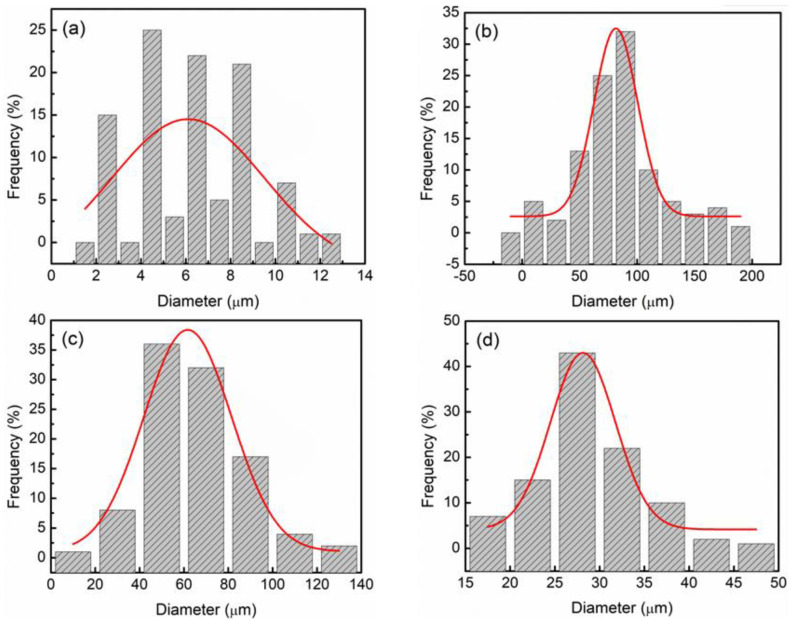
Particle size distribution histograms of the (**a**) as-obtained TiSiO_4_, (**b**) as-obtained LiAlH_4_, (**c**) post-milled LiAlH_4,_ and (**d**) LiAlH_4_ added with 10 wt% TiSiO_4_.

The particle size distribution of the TiSiO_4_, added, and undoped LiAlH_4_ samples were determined using the Image J software, as demonstrated in Figure 5. Based on the histogram shown in Figure 5a–d, the estimated average particle sizes for the as-obtained TiSiO_4_, as-obtained LiAlH_4_, post-milled LiAlH_4_, and LiAlH_4_ added with 10 wt% TiSiO_4_ were determined to be 6.10, 81.58, 61.71, and 28.13 µm, respectively. These findings demonstrated that the addition of 10 wt% TiSiO_4_ to LiAlH_4_ significantly reduced particle size. Previous studies by Ahmad et al. [22] and Cai et al. [48] stated the added LiAlH_4_ increased the rate of hydrogen diffusion, which resulted in fast dehydrogenation kinetics and low activation energy. Additionally, its smaller particle size results in a larger surface area and more grain boundaries [36,38,49].

**Figure 6:**
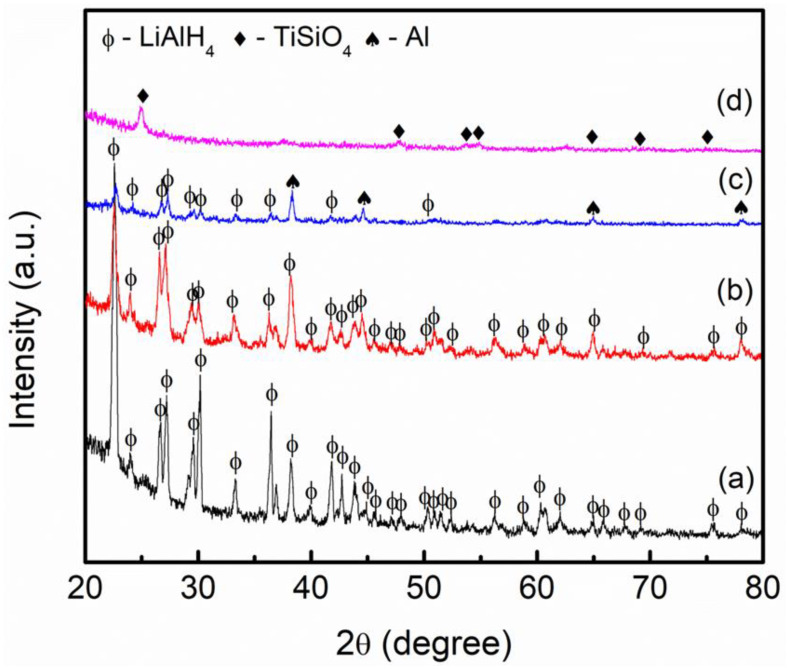
XRD results of (**a**) as-obtained LiAlH_4_, (**b**) post-milled LiAlH_4_, (**c**) LiAlH_4_ added with 10 wt% of TiSiO_4,_ and (**d**) as-obtained TiSiO_4_.

Figure 6 shows the XRD pattern of the as-obtained and post-milled LiAlH_4_, TiSiO_4_-doped LiAlH_4_ composite, and as-obtained TiSiO_4_ sample. The sample of as-obtained TiSiO_4_ shows the high purity of TiSiO_4_ [50]. For the as-obtained LiAlH_4_ sample, as shown in Figure 6a, the peaks of LiAlH_4_ are dominant, and there are no other peaks detected, proving that the as-obtained LiAlH_4_ sample is pure, as stated by the supplier. Furthermore, as shown in Figure 6b, even after the ball milling process of 1 h, LiAlH_4_ initial phase remains unchanged. According to this outcome and the findings of Sazelee et al. [15] and Balema et al. [51], LiAlH_4_ was stable even after being subjected to ball milling. Figure 6c shows the XRD pattern of the LiAlH_4_–10 wt% TiSiO_4_ composite. In addition to the majority peaks of LiAlH_4_, there is a new peak corresponding to the Al for the TiSiO_4_-doped LiAlH_4_ after 1 h milling. The appearance of Al peaks indicates that with the presence of TiSiO_4_, hydrogen is slightly released from LiAlH_4_ after milling (Equation (1)). However, the peaks that correspond to Li_3_AlH_6_ could not be detected in this pattern. Additionally, due to the small amount of TiSiO_4_ used or the fact that TiSiO_4_ became amorphous after 1 h of the milling process, the peaks of TiSiO_4_ could not be detected with an XRD pattern [39,49,52].

**Figure 7:**
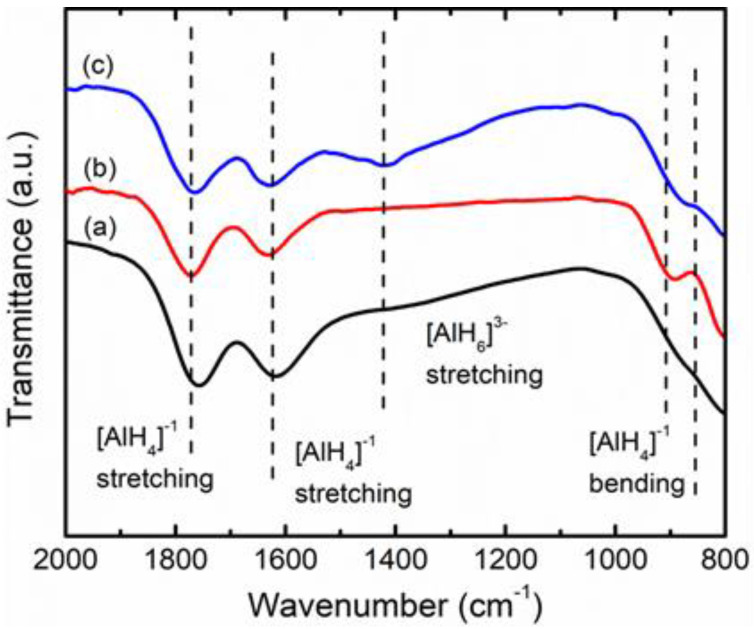
FTIR pattern of the (**a**) as-obtained LiAlH_4_, (**b**) post-milled LiAlH_4_, and (**c**) LiAlH_4_ added with 10 wt% of TiSiO_4_.

Figure 7 depicts the FTIR spectrum used to investigate the effect of TiSiO_4_ as a catalyst on the LiAlH_4_ infrared spectroscopy band and to confirm the presence of Li_3_AlH_6_ in the TiSiO_4_-doped LiAlH_4_ sample after milling. In the previous studies [15,37,53], LiAlH_4_ was represented by peaks with two bending modes between 800 and 900 cm^−1^ and two stretching modes between 1600 and 1800 cm^−1^. Figure 7a,b show these peaks in the as-obtained and post-milled LiAlH_4_ samples. After 1 h of milling (Figure 7c), a new peak at 1403 cm^−1^ was discovered in the TiSiO_4_-doped LiAlH_4_ sample. This new peak corresponds to the Al–H stretching mode of Li_3_AlH_6_. Although the peak of Li_3_AlH_6_ could not be detected in the XRD pattern (Figure 6c), the appearance of the Al–H stretching mode of Li_3_AlH_6_ in the FTIR result proves that the TiSiO_4_-doped LiAlH_4_ sample slightly released hydrogen during the milling process (Equation (1)).

**Figure 8:**
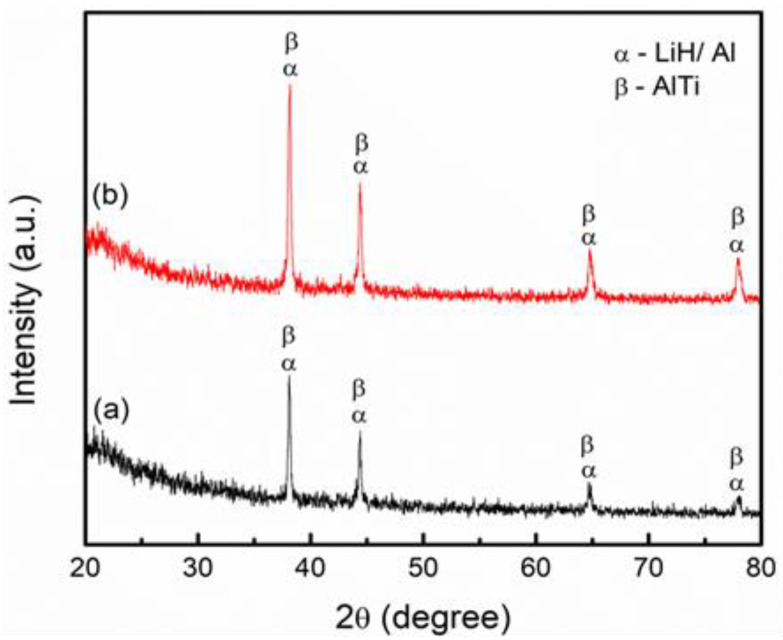
XRD pattern of the dehydrogenation at 250 °C of the added LiAlH_4_ with (**a**) 10 wt% and (**b**) 20 wt% of TiSiO_4_.

The XRD analysis helped to identify the catalytic and reaction mechanism behind the improvement of the dehydrogenation properties of TiSiO_4_-added LiAlH_4_. Figure 8 shows the results of 10 wt% and 20 wt% TiSiO_4_-added LiAlH_4_ composites after the dehydrogenation process at 250 °C. Due to the small amount of catalyst used, it may be challenging to identify the active species in the 10 wt% TiSiO_4_-added LiAlH_4_, so 20 wt% TiSiO_4_ was added to LiAlH_4_. The presence of the Al and LiH peaks in both samples (Figure 8a) indicates that LiAlH_4_ has been completely dehydrogenated (Equation (2)). In addition to Al and LiH peaks, the peaks that correspond to the AlTi species can also be detected in the dehydrogenation sample. However, the peaks that correspond to the Si or Si-containing patterns could not be observed after the dehydrogenation process. This may be because the Si or Si-containing patterns were in an amorphous state. According to this research, it was hypothesized that the enhanced dehydrogenation behaviors of LiAlH_4_ were attributed to the formation of AlTi and Si or Si-containing species. Furthermore, there were no new peaks found in the XRD patterns of the desorbed 20 wt% TiSiO_4_-added LiAlH_4_ composite (Figure 8b), which are similar to those of the 10 wt% TiSiO_4_-added LiAlH_4_ composite (Figure 8a). The peak of Al, LiH, and AlTi did not change, and no other patterns were discovered in the dehydrogenation composite, possibly because these patterns were in an amorphous state when the dehydrogenation process was complete.

Previous studies have shown that the *in-situ* emergence of AlTi helps to improve the dehydrogenation behavior of LiAlH_4_ [39,49,54]. Strong surface reactions between the Ti atom and LiAlH_4_ caused a reduction in the H binding energy. The correlation between the charge transfer change between Al and H and the rapid kinetic efficiency of LiAlH_4_ may explain the decrease in binding energy. Although the Si or Si-containing phase was not found, these species likewise play a particular role in enhancing the dehydrogenation behavior of LiAlH_4_. For example, the Si-containing species have been proven to play a significant role in enhancing the hydrogen storage properties of MgH_2_ [55]. Another study proved that SiC could enhance the hydrogen storage properties of MgH_2_ by reducing grain size and increasing defect concentration in MgH_2_ particles [56,57]. However, more research is still needed, such as using high-resolution transmission electron microscopy and X-ray photoelectron spectroscopy to clarify the exact catalytic role of TiSiO_4_ on the hydrogen storage properties of LiAlH_4_.

## 4. Conclusions

In conclusion, the addition of TiSiO_4_ improved the dehydrogenation properties of LiAlH_4_. The introduction of TiSiO_4_ to LiAlH_4_ reduced the dehydrogenation temperatures for the first and second stages (start decomposing at 92 °C and 128 °C, respectively). Dehydrogenation kinetic analysis at 90 °C revealed that after 2 h, the TiSiO_4_-added LiAlH_4_ composite was able to liberate more hydrogen (about 6.00 wt%) than the undoped LiAlH_4_ composite (less than 1.00 wt%). The *E*_A_ for hydrogen liberated from LiAlH_4_ was reduced after TiSiO_4_ was added. According to the Kissinger equation, the *E*_A_ for hydrogen liberated in the two-step dehydrogenation of post-milled LiAlH_4_ was 103 and 115 kJ/mol, respectively. The *E*_A_ were lowered to 68 and 77 kJ/mol, respectively, after milling LiAlH_4_ with 10 wt% of TiSiO_4_. The addition of 10 wt% of TiSiO_4_ also caused the LiAlH_4_ particles to shrink and become less aggregated. According to the XRD results, TiSiO_4_ significantly enhanced the dehydrogenation properties of LiAlH_4_ by forming active AlTi and Si-containing species during the heating process.

## Figures and Tables

**Table 1:** Properties of LiAlH_4_ and other complex hydrides [21,22,23,24,25].

Materials	Cost of Material ($ g^−1^)	Desorption Kinetics	Gravimetric (wt%)	Volumetric (g L^−1^)
LiAlH_4_	3.7	~0.04 wt% (within 180 min, 90 °C)	10.5	96.7
NaAlH_4_	10.8	~0.01 wt% (within 150 min, 140 °C)	7.4	94.8
KBH_4_	1.41	NA	7.4	87.1
NaNH_2_	6.19	NA	5.2	71.9

## Data Availability

The data presented in this study are available on request from the corresponding author.
